# Bis[dichlorido(5,5′-dimethyl-2,2′-bi­pyridine-κ^2^ *N*,*N*′)gold(III)] tetra­chlorido­aurate(III) dichloridooaurate(I)

**DOI:** 10.1107/S1600536809006436

**Published:** 2009-02-28

**Authors:** Selvi Karaca, Mehmet Akkurt, Nasser Safari, Vahid Amani, Orhan Büyükgüngör, Anita Abedi

**Affiliations:** a Department of Physics, Faculty of Arts and Sciences, Erciyes University, 38039 Kayseri, Turkey; b Chemistry Department, Shahid Beheshti University, GC, Tehran, Iran; c Department of Physics, Faculty of Arts and Sciences, Ondokuz Mayıs University, 55139 Samsun, Turkey; d Department of Chemistry, North Tehran Branch, Islamic Azad University, Tehran, Iran

## Abstract

The title compound, [Au^III^Cl_2_(C_12_H_12_N_2_)]_2_[Au^III^Cl_4_][Au^I^Cl_2_], contains three distinct types of Au atom. In the cation, the Au^III^ atom is four-coordinated in a distorted square-planar arrangement by an *N*,*N*′-bidentate 5,5′-dimethyl-2,2′-bipyridine ligand and two terminal Cl atoms. In the [AuCl_4_]^−^ anion, the centrosymmetric Au^III^ atom has a square-planar coordination. The centrosymmetric [AuCl_2_]^−^ anion is linear. Intra- and inter­molecular C—H⋯Cl hydrogen bonds help to establish the conformation and packing.

## Related literature

For related stuctures, see: Abbate *et al.* (2000 ▶); Adams & Strähle (1982 ▶); Ahmadi, Amani & Khavasi (2008 ▶); Ahmadi, Dehghan, Amani & Khavasi (2008 ▶); Bjernemose *et al.* (2004 ▶); Hayoun *et al.* (2006 ▶); Hollis & Lippard (1983 ▶); McInnes *et al.* (1995 ▶); Yıldırım *et al.* (2008 ▶). 
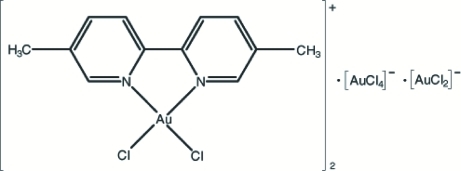


## Experimental

### 

#### Crystal data

[AuCl_2_(C_12_H_12_N_2_)]_2_[AuCl_4_][AuCl_2_]
*M* *_r_* = 1510.86
Triclinic, 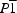

*a* = 9.0698 (4) Å
*b* = 10.0886 (4) Å
*c* = 11.1678 (5) Å
α = 91.155 (4)°
β = 108.148 (4)°
γ = 111.344 (3)°
*V* = 894.09 (7) Å^3^
*Z* = 1
Mo *K*α radiation
μ = 17.13 mm^−1^
*T* = 295 K
0.41 × 0.28 × 0.08 mm

#### Data collection

Stoe IPDS-2 diffractometer
Absorption correction: integration (*X-RED32*; Stoe & Cie, 2002 ▶) *T*_min_ = 0.054, *T*_max_ = 0.341
9898 measured reflections
3651 independent reflections
3193 reflections with *I* > 2σ(*I*)
*R*_int_ = 0.059

#### Refinement

*R*[*F* ^2^ > 2σ(*F* ^2^)] = 0.042
*wR*(*F* ^2^) = 0.112
*S* = 1.05
3651 reflections
191 parameters
H-atom parameters constrained
Δρ_max_ = 1.64 e Å^−3^
Δρ_min_ = −1.91 e Å^−3^

### 

Data collection: *X-AREA* (Stoe & Cie, 2002 ▶); cell refinement: *X-AREA*; data reduction: *X-RED32* (Stoe & Cie, 2002 ▶); program(s) used to solve structure: *SIR97* (Altomare *et al.*, 1999 ▶); program(s) used to refine structure: *SHELXL97* (Sheldrick, 2008 ▶); molecular graphics: *ORTEP-3 for Windows* (Farrugia, 1997 ▶); software used to prepare material for publication: *WinGX* (Farrugia, 1999 ▶).

## Supplementary Material

Crystal structure: contains datablocks global, I. DOI: 10.1107/S1600536809006436/hb2902sup1.cif

Structure factors: contains datablocks I. DOI: 10.1107/S1600536809006436/hb2902Isup2.hkl

Additional supplementary materials:  crystallographic information; 3D view; checkCIF report

## Figures and Tables

**Table 1:** Selected bond lengths (Å)

Au1—N1	2.028 (9)
Au1—N2	2.027 (7)
Au1—Cl1	2.252 (3)
Au1—Cl2	2.262 (3)
Au2—Cl3	2.246 (5)
Au2—Cl4	2.261 (3)
Au3—Cl5	2.248 (3)

**Table 2:** Hydrogen-bond geometry (Å, °)

*D*—H⋯*A*	*D*—H	H⋯*A*	*D*⋯*A*	*D*—H⋯*A*
C1—H1⋯Cl1	0.93	2.59	3.203 (11)	124
C8—H8⋯Cl2^i^	0.93	2.75	3.666 (12)	169
C11—H11⋯Cl2	0.93	2.64	3.233 (11)	122

Symmetry code: (i) 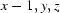.

**Fig. 1.**
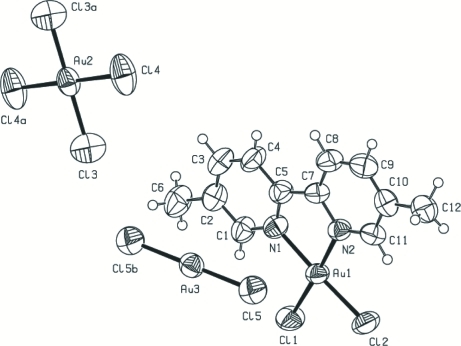
The molecular structure of (I) showing 50% probability displacement ellipsoids for the non-hydrogen atoms. Symmetry code suffixes: (a) –x, –y, –z; (b) 2–x, 1–y, 1–z.

**Fig. 2.**
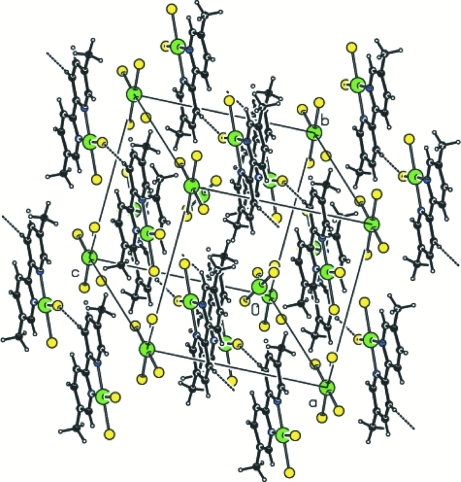
A general view of the packing and hydrogen bonding interactions in (I).

## supplementary crystallographic information

### Comment

Recently, we reported the synthesis and crystal structure of the [Au(dtbpy)Cl_2_][AuCl_4_].CH_3_CN, (II), (Yıldırım *et al.*, 2008) [where dtbpy is 4,4'-di-*tert*-butyl-2,2'-bipyridine]. There are several Au^III^ complexes, with formula, [AuCl_2_ (N—N)]*X*, such as [AuCl_2_(bipy)][BF_4_], (III), (McInnes *et al.*, 1995), [AuCl_2_(bipy)](NO_3_), (IV), (Bjernemose *et al.*, 2004), [AuCl_2_(bipy)][AuBr_4_], (V), (Hayoun *et al.*, 2006), [AuCl_2_(dmphen)][AuCl_4_], (VI), (Ahmadi, Amani *et al.*, 2008) and [AuCl_2_ (phen)]Cl.H_2_O, (VII), (Abbate *et al.*, 2000) [where bipy is 2,2'-bipyridine, dmphen is 4,7-diphenyl-1,10-phenanthroline and phen is 1,10-phenanthroline] have been synthesized and characterized by single-crystal X-ray diffraction methods. Two Au^III^ complexes with formula, [AuCl_2_*L*_2_]*X*, [AuCl_2_(py)_2_][AuCl_4_], (VIII), and [AuCl_2_(py)_2_]Cl.H_2_O, (IX), (Adams & Strähle 1982) [py is pyridine] and two mixed-valence Au^I^—Au^III^ complexes, [Au(terpy)Cl]_2_[AuCl_2_]_3_[AuCl_4_], (*X*), (Hollis & Lippard, 1983) and [Au(dmpy)_2_][AuCl_4_], (XI), (Ahmadi, Dehghan *et al.*, 2008) [where terpy is 2,2',2''-terpyridine and dmpy is 2,6-dimethylpyridine] have been synthesized and characterized by single-crystal X-ray diffraction methods. We report herein the synthesis and crystal structure of the title compound (I).

In the asymmetric unit of the title compound (I), (Fig. 1), there are one cation and two half-anions. In the cation, the Au^III^ atom is four-coordinated in a distorted square-planar configuration by two N atoms from the ligand and two terminal Cl atoms. In the anion AuCl_4_, the Au^III^ atom has a square-planar coordination. The anion AuCl_2_ is linear. In the cation, the Au—Cl and Au—N bond lengths and angles (Table 1) are in good agreement with the corresponding values in (II), (III), (IV), (V), (VI), (VII), (VIII), (IX), (*X*) and, (XI). In the anion, the Au—Cl bond lengths and angles (Table 1) are normal.

Intra and intermolecular C—H···Cl hydrogen bonding interactions (Table 2) stabilize the molecular conformation and the packing arrangement (Fig. 2).

### Experimental

A solution of 5,5'-dimethyl-2,2'-bipyridine (0.20 g, 1.09 mmol) in ethanol (20 ml) was added to a solution of HAuCl_3_.3H_2_O, (0.37 g, 1.09 mmol) in acetonitrile (20 ml) and the resulting yellow solution was stirred for 10 min at 313 K. Then, it was left to evaporate slowly at room temperature. After one week, yellow prismatic crystals of (I) were isolated (yield 0.28 g, 72.8%; m.p. 553 K).

### Refinement

All H-atoms were placed in calculated positions with C—H = 0.93 Å and C—H 0.96 Å, and were included in the refinement in the riding model approximation, with *U*_iso_(H) = 1.2*U*_eq_ (ring C) and *U*_iso_(H) = 1.5*U*_eq_ (methyl C). In the final Fourier map, the highest and deepest peaks were located 0.91 and 0.81 Å from atom Au1, respectively.

### Crystal data

**Table:**

[AuCl_2_(C_12_H_12_N_2_)]_2_[AuCl_4_][AuCl_2_]	*Z* = 1
*M**_r_* = 1510.86	*F*(000) = 682
Triclinic, *P*1	*D*_x_ = 2.806 Mg m^−^^3^
Hall symbol: -P 1	Mo *K*α radiation, λ = 0.71073 Å
*a* = 9.0698 (4) Å	Cell parameters from 16778 reflections
*b* = 10.0886 (4) Å	θ = 1.9–28.0°
*c* = 11.1678 (5) Å	µ = 17.13 mm^−^^1^
α = 91.155 (4)°	*T* = 295 K
β = 108.148 (4)°	Prism, yellow
γ = 111.344 (3)°	0.41 × 0.28 × 0.08 mm
*V* = 894.09 (7) Å^3^	

### Data collection

**Table:**

Stoe IPDS-2 diffractometer	3651 independent reflections
Radiation source: sealed X-ray tube, 12 x 0.4 mm long-fine focus	3193 reflections with *I* > 2σ(*I*)
plane graphite	*R*_int_ = 0.059
Detector resolution: 6.67 pixels mm^-1^	θ_max_ = 26.5°, θ_min_ = 1.9°
ω scans	*h* = −10→11
Absorption correction: integration (*X-RED32*; Stoe & Cie, 2002)	*k* = −12→12
*T*_min_ = 0.054, *T*_max_ = 0.341	*l* = −14→14
9898 measured reflections	

### Refinement

**Table:**

Refinement on *F*^2^	Secondary atom site location: difference Fourier map
Least-squares matrix: full	Hydrogen site location: inferred from neighbouring sites
*R*[*F*^2^ > 2σ(*F*^2^)] = 0.042	H-atom parameters constrained
*wR*(*F*^2^) = 0.112	*w* = 1/[σ^2^(*F*_o_^2^) + (0.0703*P*)^2^ + 0.309*P*] where *P* = (*F*_o_^2^ + 2*F*_c_^2^)/3
*S* = 1.05	(Δ/σ)_max_ = 0.001
3651 reflections	Δρ_max_ = 1.64 e Å^−^^3^
191 parameters	Δρ_min_ = −1.91 e Å^−^^3^
0 restraints	Extinction correction: *SHELXL97* (Sheldrick, 2008), Fc^*^=KFc[1+0.001XFc^2^Λ^3^/sin(2θ)]^-1/4^
Primary atom site location: structure-invariant direct methods	Extinction coefficient: 0.0042 (5)

### Special details

**Table:**

Geometry. Bond distances, angles *etc*. have been calculated using the rounded fractional coordinates. All su's are estimated from the variances of the (full) variance-covariance matrix. The cell e.s.d.'s are taken into account in the estimation of distances, angles and torsion angles
Refinement. Refinement on *F*^2^ for ALL reflections except those flagged by the user for potential systematic errors. Weighted *R*-factors *wR* and all goodnesses of fit *S* are based on *F*^2^, conventional *R*-factors *R* are based on *F*, with *F* set to zero for negative *F*^2^. The observed criterion of *F*^2^ > σ(*F*^2^) is used only for calculating -*R*-factor-obs *etc*. and is not relevant to the choice of reflections for refinement. *R*-factors based on *F*^2^ are statistically about twice as large as those based on *F*, and *R*-factors based on ALL data will be even larger.

### Fractional atomic coordinates and isotropic or equivalent isotropic displacement parameters (Å^2^)

**Table:**

	*x*	*y*	*z*	*U*_iso_*/*U*_eq_	
Au1	1.23403 (3)	0.77774 (3)	0.26312 (3)	0.0544 (1)	
Cl1	1.2859 (3)	0.5914 (3)	0.1987 (3)	0.0790 (9)	
Cl2	1.5117 (3)	0.9173 (3)	0.3196 (3)	0.0782 (9)	
N1	0.9831 (9)	0.6658 (8)	0.2201 (7)	0.060 (2)	
N2	1.1693 (8)	0.9371 (7)	0.3140 (6)	0.0528 (19)	
C1	0.9039 (11)	0.5243 (9)	0.1758 (9)	0.066 (3)	
C2	0.7317 (12)	0.4506 (10)	0.1500 (9)	0.069 (3)	
C3	0.6467 (11)	0.5303 (11)	0.1758 (10)	0.076 (3)	
C4	0.7298 (11)	0.6737 (11)	0.2244 (10)	0.074 (3)	
C5	0.8990 (11)	0.7414 (9)	0.2443 (8)	0.060 (3)	
C6	0.6455 (15)	0.2922 (11)	0.1035 (13)	0.093 (4)	
C7	1.0005 (10)	0.8939 (9)	0.2956 (8)	0.056 (3)	
C8	0.9407 (12)	0.9933 (11)	0.3240 (10)	0.069 (3)	
C9	1.0501 (13)	1.1320 (11)	0.3714 (11)	0.076 (3)	
C10	1.2194 (13)	1.1753 (9)	0.3894 (10)	0.070 (3)	
C11	1.2737 (11)	1.0742 (9)	0.3607 (8)	0.060 (3)	
C12	1.3426 (14)	1.3307 (10)	0.4439 (12)	0.082 (4)	
Au2	0.00000	0.00000	0.00000	0.0681 (2)	
Cl3	0.2497 (5)	−0.0056 (5)	0.0129 (5)	0.1187 (16)	
Cl4	0.1272 (6)	0.2391 (3)	0.0717 (3)	0.1093 (12)	
Au3	1.00000	0.50000	0.50000	0.0654 (2)	
Cl5	1.2288 (4)	0.7061 (3)	0.5439 (3)	0.0836 (7)*	
H1	0.96620	0.47370	0.16190	0.0790*	
H3	0.53150	0.48610	0.16010	0.0910*	
H4	0.67160	0.72540	0.24380	0.0880*	
H6A	0.62170	0.24230	0.17190	0.1400*	
H6B	0.71750	0.25990	0.07430	0.1400*	
H6C	0.54210	0.27280	0.03460	0.1400*	
H8	0.82660	0.96600	0.31100	0.0820*	
H9	1.00990	1.19880	0.39200	0.0910*	
H11	1.38780	1.10100	0.37400	0.0730*	
H12A	1.38440	1.34120	0.53520	0.1240*	
H12B	1.28570	1.39460	0.41790	0.1240*	
H12C	1.43510	1.35400	0.41270	0.1240*	

### Atomic displacement parameters (Å^2^)

**Table:**

	*U*^11^	*U*^22^	*U*^33^	*U*^12^	*U*^13^	*U*^23^
Au1	0.0476 (2)	0.0555 (2)	0.0651 (2)	0.0246 (1)	0.0206 (1)	0.0069 (1)
Cl1	0.0748 (14)	0.0681 (12)	0.1089 (19)	0.0387 (10)	0.0390 (13)	0.0006 (12)
Cl2	0.0476 (10)	0.0728 (13)	0.113 (2)	0.0220 (9)	0.0284 (11)	0.0031 (13)
N1	0.053 (3)	0.062 (4)	0.059 (4)	0.019 (3)	0.017 (3)	0.002 (3)
N2	0.051 (3)	0.060 (3)	0.053 (4)	0.025 (3)	0.021 (3)	0.009 (3)
C1	0.062 (5)	0.057 (4)	0.074 (6)	0.015 (4)	0.027 (4)	0.002 (4)
C2	0.067 (5)	0.066 (5)	0.062 (5)	0.015 (4)	0.022 (4)	0.000 (4)
C3	0.051 (5)	0.080 (6)	0.079 (6)	0.007 (4)	0.023 (4)	0.003 (5)
C4	0.043 (4)	0.072 (5)	0.091 (7)	0.007 (4)	0.021 (4)	0.005 (5)
C5	0.057 (4)	0.066 (5)	0.061 (5)	0.025 (4)	0.025 (4)	0.011 (4)
C6	0.077 (7)	0.072 (6)	0.107 (9)	0.005 (5)	0.031 (6)	−0.010 (6)
C7	0.050 (4)	0.062 (4)	0.060 (5)	0.028 (3)	0.018 (3)	0.006 (4)
C8	0.061 (5)	0.078 (5)	0.079 (6)	0.036 (4)	0.030 (4)	0.012 (5)
C9	0.085 (6)	0.071 (5)	0.094 (7)	0.047 (5)	0.040 (5)	0.009 (5)
C10	0.084 (6)	0.054 (4)	0.071 (6)	0.027 (4)	0.026 (5)	0.011 (4)
C11	0.060 (5)	0.060 (4)	0.062 (5)	0.031 (4)	0.014 (4)	0.006 (4)
C12	0.087 (7)	0.057 (5)	0.103 (8)	0.031 (4)	0.029 (6)	0.007 (5)
Au2	0.0810 (3)	0.0527 (3)	0.0547 (3)	0.0170 (2)	0.0130 (2)	0.0067 (2)
Cl3	0.098 (2)	0.133 (3)	0.127 (3)	0.052 (2)	0.033 (2)	0.018 (2)
Cl4	0.160 (3)	0.0549 (12)	0.0797 (18)	0.0144 (15)	0.0305 (19)	0.0013 (11)
Au3	0.0724 (3)	0.0670 (3)	0.0672 (3)	0.0335 (2)	0.0296 (2)	0.0161 (2)

### Geometric parameters (Å, °)

**Table:**

Au1—N1	2.028 (9)	C5—C7	1.466 (12)
Au1—N2	2.027 (7)	C7—C8	1.375 (15)
Au1—Cl1	2.252 (3)	C8—C9	1.362 (15)
Au1—Cl2	2.262 (3)	C9—C10	1.380 (18)
Au2—Cl4^i^	2.261 (3)	C10—C11	1.355 (15)
Au2—Cl3^i^	2.246 (5)	C10—C12	1.530 (14)
Au2—Cl3	2.246 (5)	C1—H1	0.9300
Au2—Cl4	2.261 (3)	C3—H3	0.9300
Au3—Cl5^ii^	2.248 (3)	C4—H4	0.9300
Au3—Cl5	2.248 (3)	C6—H6B	0.9600
N1—C1	1.343 (12)	C6—H6A	0.9600
N1—C5	1.334 (13)	C6—H6C	0.9600
N2—C7	1.375 (13)	C8—H8	0.9300
N2—C11	1.339 (11)	C9—H9	0.9300
C1—C2	1.393 (15)	C11—H11	0.9300
C2—C3	1.380 (16)	C12—H12C	0.9600
C2—C6	1.496 (14)	C12—H12A	0.9600
C3—C4	1.369 (15)	C12—H12B	0.9600
C4—C5	1.375 (15)		
			
Au1···Cl3^iii^	3.588 (5)	N1···C7	2.368 (12)
Au1···Cl5	3.243 (3)	N1···Cl1	3.173 (9)
Au2···C7^iv^	3.490 (8)	N2···Cl2	3.165 (9)
Au2···C7^v^	3.490 (8)	N2···N1	2.619 (10)
Au3···C1	3.486 (9)	N2···C5	2.396 (12)
Au3···C1^ii^	3.486 (9)	C1···Au3	3.486 (9)
Cl1···Cl4^vi^	3.394 (4)	C1···Au3	3.486 (9)
Cl1···N1	3.173 (9)	C3···Cl1^viii^	3.625 (12)
Cl1···C1	3.203 (11)	C5···Cl4^iv^	3.477 (9)
Cl1···Cl2	3.166 (4)	C6···Cl3	3.564 (14)
Cl1···C3^vi^	3.625 (12)	C7···Au2^iv^	3.490 (8)
Cl2···N2	3.165 (9)	C7···Au2^iii^	3.490 (8)
Cl2···C11	3.233 (11)	C8···Cl3^iv^	3.644 (12)
Cl2···Cl1	3.166 (4)	C10···Cl4^iii^	3.513 (11)
Cl2···C11^vii^	3.476 (9)	C11···Cl2^vii^	3.476 (9)
Cl2···Cl5^vii^	3.650 (4)	C12···C12^xi^	3.448 (15)
Cl3···C6	3.564 (14)	C4···H8	2.8100
Cl3···C8^iv^	3.644 (12)	C8···H4	2.7900
Cl3···Cl4	3.185 (7)	H1···Cl1	2.5900
Cl3···Au1^v^	3.588 (5)	H1···H6B	2.3900
Cl3···Cl4^i^	3.188 (7)	H3···Cl1^viii^	2.9300
Cl4···Cl3^i^	3.188 (7)	H3···H6C	2.5900
Cl4···C10^v^	3.513 (11)	H4···C8	2.7900
Cl4···C5^iv^	3.477 (9)	H4···Cl1^viii^	3.1200
Cl4···Cl3	3.185 (7)	H4···Cl2^viii^	3.0500
Cl4···Cl1^viii^	3.394 (4)	H4···H8	2.2800
Cl4···N1^iv^	3.367 (8)	H6A···Cl5^ii^	2.9900
Cl5···Au1	3.243 (3)	H6B···H1	2.3900
Cl5···Cl2^vii^	3.650 (4)	H6B···Cl3^ix^	2.8700
Cl1···H4^vi^	3.1200	H6C···H3	2.5900
Cl1···H1	2.5900	H6C···Cl3	3.0200
Cl1···H3^vi^	2.9300	H8···H4	2.2800
Cl2···H8^vi^	2.7500	H8···C4	2.8100
Cl2···H11	2.6400	H8···Cl2^viii^	2.7500
Cl2···H4^vi^	3.0500	H9···H12B	2.4800
Cl3···H6C	3.0200	H9···Cl5^x^	2.9300
Cl3···H6B^ix^	2.8700	H11···Cl2	2.6400
Cl5···H11^vii^	3.1200	H11···H12C	2.4300
Cl5···H6A^ii^	2.9900	H11···Cl5^vii^	3.1200
Cl5···H9^x^	2.9300	H12B···H9	2.4800
N1···Cl4^iv^	3.367 (8)	H12C···H11	2.4300
N1···N2	2.619 (10)		
			
Cl1—Au1—Cl2	89.07 (11)	C7—C8—C9	119.4 (11)
Cl1—Au1—N1	95.5 (2)	C8—C9—C10	121.3 (11)
Cl1—Au1—N2	175.8 (2)	C11—C10—C12	121.1 (11)
Cl2—Au1—N1	175.3 (2)	C9—C10—C11	117.6 (9)
Cl2—Au1—N2	95.0 (2)	C9—C10—C12	121.3 (10)
N1—Au1—N2	80.5 (3)	N2—C11—C10	122.5 (10)
Cl3^i^—Au2—Cl4^i^	89.95 (19)	C2—C1—H1	119.00
Cl3—Au2—Cl4^i^	90.05 (19)	N1—C1—H1	119.00
Cl3—Au2—Cl4	89.95 (19)	C2—C3—H3	120.00
Cl3—Au2—Cl3^i^	180.00	C4—C3—H3	120.00
Cl3^i^—Au2—Cl4	90.05 (19)	C3—C4—H4	120.00
Cl4—Au2—Cl4^i^	180.00	C5—C4—H4	120.00
Cl5—Au3—Cl5^ii^	180.00	C2—C6—H6A	109.00
Au1—N1—C1	124.0 (7)	C2—C6—H6B	109.00
Au1—N1—C5	115.3 (6)	C2—C6—H6C	109.00
C1—N1—C5	120.7 (9)	H6B—C6—H6C	109.00
Au1—N2—C11	126.2 (7)	H6A—C6—H6B	109.00
Au1—N2—C7	113.9 (5)	H6A—C6—H6C	109.00
C7—N2—C11	120.0 (8)	C7—C8—H8	120.00
N1—C1—C2	122.3 (10)	C9—C8—H8	120.00
C3—C2—C6	121.8 (11)	C10—C9—H9	119.00
C1—C2—C3	116.3 (9)	C8—C9—H9	119.00
C1—C2—C6	121.8 (10)	N2—C11—H11	119.00
C2—C3—C4	120.9 (10)	C10—C11—H11	119.00
C3—C4—C5	120.1 (10)	C10—C12—H12A	110.00
N1—C5—C4	119.7 (8)	C10—C12—H12B	109.00
N1—C5—C7	115.4 (9)	C10—C12—H12C	109.00
C4—C5—C7	124.8 (9)	H12A—C12—H12B	109.00
N2—C7—C5	115.0 (8)	H12A—C12—H12C	109.00
N2—C7—C8	119.3 (8)	H12B—C12—H12C	109.00
C5—C7—C8	125.8 (9)		
			
Cl1—Au1—N1—C1	3.4 (7)	C11—N2—C7—C8	0.9 (12)
N2—Au1—N1—C1	−177.7 (8)	N1—C1—C2—C3	−1.7 (14)
Cl1—Au1—N1—C5	−179.5 (6)	N1—C1—C2—C6	−178.6 (10)
N2—Au1—N1—C5	−0.7 (6)	C6—C2—C3—C4	176.7 (10)
Cl2—Au1—N2—C7	−179.0 (5)	C1—C2—C3—C4	−0.2 (15)
N1—Au1—N2—C7	0.0 (6)	C2—C3—C4—C5	2.1 (16)
Cl2—Au1—N2—C11	1.5 (7)	C3—C4—C5—C7	180.0 (9)
N1—Au1—N2—C11	−179.5 (7)	C3—C4—C5—N1	−2.2 (14)
Au1—N1—C1—C2	178.6 (7)	C4—C5—C7—N2	176.7 (8)
C5—N1—C1—C2	1.7 (14)	C4—C5—C7—C8	−4.2 (15)
Au1—N1—C5—C4	−176.8 (7)	N1—C5—C7—N2	−1.2 (11)
C1—N1—C5—C4	0.4 (13)	N1—C5—C7—C8	177.9 (9)
Au1—N1—C5—C7	1.2 (10)	C5—C7—C8—C9	180.0 (9)
C1—N1—C5—C7	178.4 (8)	N2—C7—C8—C9	−0.9 (14)
Au1—N2—C11—C10	178.3 (7)	C7—C8—C9—C10	1.1 (16)
C7—N2—C11—C10	−1.1 (13)	C8—C9—C10—C11	−1.3 (16)
Au1—N2—C7—C8	−178.6 (7)	C8—C9—C10—C12	−179.4 (10)
Au1—N2—C7—C5	0.6 (9)	C12—C10—C11—N2	179.4 (9)
C11—N2—C7—C5	−179.9 (7)	C9—C10—C11—N2	1.3 (14)

Symmetry codes: (i) −*x*, −*y*, −*z*; (ii) −*x*+2, −*y*+1, −*z*+1; (iii) *x*+1, *y*+1, *z*; (iv) −*x*+1, −*y*+1, −*z*; (v) *x*−1, *y*−1, *z*; (vi) *x*+1, *y*, *z*; (vii) −*x*+3, −*y*+2, −*z*+1; (viii) *x*−1, *y*, *z*; (ix) −*x*+1, −*y*, −*z*; (x) −*x*+2, −*y*+2, −*z*+1; (xi) −*x*+3, −*y*+3, −*z*+1.

### Hydrogen-bond geometry (Å, °)

**Table:**

*D*—H···*A*	*D*—H	H···*A*	*D*···*A*	*D*—H···*A*
C1—H1···Cl1	0.93	2.59	3.203 (11)	124
C8—H8···Cl2^viii^	0.93	2.75	3.666 (12)	169
C11—H11···Cl2	0.93	2.64	3.233 (11)	122

Symmetry codes: (viii) *x*−1, *y*, *z*.
